# Alternatives to Low Molecular Weight Heparin for Anticoagulation in Pregnant Women with Mechanical Heart Valves in Middle-Income Countries: A Cohort Study

**DOI:** 10.5334/gh.1011

**Published:** 2021-10-13

**Authors:** Anish Keepanasseril, Ajith Ananthakrishna Pillai, Jyoti Baghel, Swaraj Nandini Pande, Nivedita Mondal, Hemachandren Munuswamy, Pankaj Kundra, Rohan D’Souza

**Affiliations:** 1 Departments of Obstetrics and Gynaecology, Jawaharlal Institute of Post-graduate Medical Education and Research (JIPMER), Puducherry, IN; 2 Departments of Cardiology, Jawaharlal Institute of Post-graduate Medical Education and Research (JIPMER), Puducherry, IN; 3 Departments of Neonatology, Jawaharlal Institute of Post-graduate Medical Education and Research (JIPMER), Puducherry, IN; 4 Departments of Cardiothoracic Vascular Surgery, Jawaharlal Institute of Post-graduate Medical Education and Research (JIPMER), Puducherry, IN; 5 Departments of Anaesthesiology and Critical Care, Jawaharlal Institute of Post-graduate Medical Education and Research (JIPMER), Puducherry, IN; 6 Division of Maternal-Fetal Medicine, Department of Obstetrics and Gynaecology, Sinai Health System, University of Toronto, Toronto, CA; 7 Lunenfeld-Tanenbaum Research Institute, Mount Sinai Hospital, Toronto, CA

**Keywords:** Mechanical heart valves, pregnancy, anticoagulation, warfarin, heparin, sequential treatment

## Abstract

**Objective:**

To compare cardiac complications and pregnancy outcomes in women with mechanical heart valves (MHVs) on two different anticoagulation regimens in a middle-income country.

**Methods:**

We conducted a retrospective cohort study comparing outcomes in pregnant women with MHVs that received vitamin K antagonists (VKAs) throughout pregnancy versus sequential anticoagulation (heparins in the first trimester and peripartum period and VKAs for the remainder of pregnancy), at a tertiary centre in South India, from January 2011 to August 2020.

**Results:**

We identified 138 pregnancies in 121 women, of whom 32 received VKAs while 106 were on sequential anticoagulation. There were no differences between groups with regard to maternal deaths [0 vs. 6 (5.7%), p = 0.34], thromboembolic events [2 (6.3%) vs. 15 (14.2%), p = 0.36], haemorrhagic complications [4 (12.5%) vs. 12 (11.3%), p = 0.85], cardiac events [1 (3.1% vs. 17 (16%), p = 0.07], spontaneous miscarriages [5 (15.6%) vs. 13 (12.3%), p = 0.62], stillbirths [0 vs. 5 (5.4%), p = 0.581] or neonatal deaths [2 (8.7%) vs. 1 (1.1%), p = 0.11]. Both cases of warfarin embryopathy received >5 mg warfarin in the first trimester. Thromboembolic events were associated with subtherapeutic doses of heparin in the first and third trimesters and the early postpartum period. Fetal growth restriction and preterm birth complicated 34 (29.3%) and 26 (22.4%) pregnancies respectively.

**Conclusion:**

Pregnancy complications associated with MHVs in middle-income countries may be reduced by multidisciplinary surveillance, avoiding first-trimester warfarin if daily doses >5 mg and ensuring therapeutic levels of heparin during bridging in the first and third trimesters and peripartum period. Administration of low-dose aspirin should be considered as this may prevent placentally-mediated complications of pregnancy.

**Highlights:**

- Pregnancy complications associated with MHVs in LMICs may be reduced by multidisciplinary surveillance, avoiding first-trimester warfarin if the daily dose is >5 mg, ensuring therapeutic levels of heparin in the first trimester and peripartum period.
- Placentally-mediated complications of pregnancy can be prevented by administering low-dose aspirin.
- Vitamin K antagonists or sequential regimen can be used as suitable alternatives to LMWH for anticoagulation in pregnant women with MHVs.

## Introduction

Low- and middle-income countries (LMICs) account for two-thirds of people living with rheumatic valvular heart disease [1,2]. The benefits of mechanical heart valves (MHVs) for valve replacement in LMICs, which include longer durability and lower-reoperation rates [3,4], must be weighed against the high risks of thromboembolic complications and need for life-long anticoagulation with vitamin K antagonists (VKAs) [3]. An additional consideration in women of reproductive age is the prothrombotic state of pregnancy and the teratogenic effects of VKAs, especially in high doses, which present unique concerns in regard to managing anticoagulation.

To date, there is no consensus regarding the ideal anticoagulation regimen in these women [5,6,7,8]. Oral VKAs are associated with a lower thrombotic risk than parenterally-administered heparins, but can cross the placenta resulting in pregnancy loss and fetal malformations; while heparins which do not traverse the placenta result in lower risks to the fetus, albeit with a higher maternal risk of thrombosis and death [9]. While the use of low molecular weight heparin (LMWH) is gaining popularity in high-resource settings [10], their use in LMICs is challenging for a number of reasons, which include women presenting late in pregnancy for antenatal care, low compliance to parenteral injections, challenges with monitoring of anticoagulant activity and cessation of anticoagulation due to high costs. For these reasons, the two commonly used anticoagulant strategies in LMICs are VKAs throughout pregnancy, and sequential treatment – heparins in the first trimester and VKAs thereafter, which theoretically confers maximum maternal benefit while minimizing fetal risk. The aim of this study was to assess maternal and perinatal outcomes in women with MHVs with the use of these two anticoagulation regimens in a tertiary referral centre in a middle-income country.

## Material and Methods

### Study design and setting

This retrospective cohort study was conducted at Jawaharlal institute of Postgraduate Medical Education & Research (JIPMER), Puducherry, India. This hospital with 16,000–17,000 births per year is the referral centre for high-risk pregnancies from Puducherry and neighbouring districts of three states, mainly catering to rural areas.

### Study population

We included all pregnancies in women with MHVs, between January 2011 and August 2020. This study includes pregnancies reported in an earlier study [11], which did not comment on anticoagulation. The study protocol and waiver of consent for gathering retrospective data, was approved by JIPMER’s Institute Ethics Committee. Patients seen after August 2015 were recruited prospectively after obtaining informed consent.

Demographic characteristics of the participants, New York Heart Association (NYHA) functional class, medical history, details of the MHV were in terms of type, site and number of replaced valves, and use of anticoagulation and other medications were noted.

### Antenatal management of pregnant women with MHVs

All pregnancies are managed by a multidisciplinary team comprising high-risk obstetricians, cardiologists, obstetric-anaesthesiologists, neonatologists and cardiothoracic surgeons. The first antenatal visit involved history-taking, baseline cardiac and obstetric evaluation, electrocardiography and echocardiography. Patients are advised monthly cardiology follow-up where patients are assessed for valve function, signs of clinical deterioration or cardiac decompensation. Obstetric follow-up continues 2-weekly until 32 weeks, and weekly thereafter until 36 weeks, wherein patients are assessed for the development of obstetric complications and fetal growth monitoring. All patients receive an anatomy ultrasound at 16–20 weeks to rule out congenital malformations, and a fetal growth scan at 30–32 weeks. In case of suspected of fetal growth restriction, ultrasound scans for fetal growth and Doppler assessment are repeated as frequently as clinically indicated.

### Management of anticoagulation (Interventions and Comparators)

If the first visit is prior to 8 weeks’ gestation, counselling regarding the two anticoagulation options is undertaken. Patients choosing sequential anticoagulation are admitted, platelet counts are assessed, and unfractionated hepartin (UFH) is commenced (15,000–20,000 U per day in 3–4 divided doses) with the aim of achieving a target activated partial thromboplastin time (aPTT) 2–2.5 times the control value. Further monitoring and titration are performed on an outpatient basis till 12 weeks’ gestation, when VKAs are recommenced. We do not use LMWH, although patients referred from other centres on LMWH are maintained on weight-adjusted doses, as anti-Xa assays are unavailable at our centre. If patients opt to continue VKAs or if there are contraindications to using UFH, the dose of VKA is adjusted to achieve an international normalized ratio (INR) target between 2.5–3.5. At each clinic visit, doses of VKA are adjusted to maintain target INR levels. Those with previous history of thromboembolism or atrial fibrillation are also started on low-dose aspirin (150 mg per day). Regardless of the anticoagulant used in the first trimester, all patients receive VKAs after 12 weeks’ gestation. At 36–37 weeks’ gestation, patients are admitted for bridging to UFH, considering the risk of labour and the risk from prolonged action of oral anticoagulation to the fetus due to delayed clearance from the fetal circulation secondary to immature fetal liver [12,13].

### Peripartum management

The mode of delivery is determined by obstetric indications. Women on VKAs presenting in labour undergo caesarean deliveries on account of the fetal bleeding risk from a vaginal birth. Labour is managed in an obstetric intensive care unit (ICU) to enable continuous maternal and fetal monitoring. All women receive infective endocarditis prophylaxis and active management of third stage of labour to reduce bleeding [14]. Care is continued in the ICU for 24–48 hours after delivery. In the absence of bleeding, UFH is commenced 6–8 hours after delivery. If there are no contraindications, pre-pregnancy dose of VKA is recommenced alongside UFH, 48 hours following delivery, Once the INR target of 2.5–3.5 is attained, UFH is stopped and anticoagulation is managed on VKA alone. Prior to discharge from hospital, patients are given contraceptive advice and follow up is arranged in the cardiology clinic.

### Outcomes

Primary (maternal) outcomes included (1) maternal death, (2) thromboembolic events (valvular and extra-valvular), (3) haemorrhagic complications including postpartum haemorrhage (blood loss ≥500 mL after vaginal birth or ≥1000 mL after caesarean delivery) or wound hematomas, and (4) cardiac complications such as heart failure (based on the clinical signs and symptoms, peripheral oedema and/or echocardiographic features of abnormal cardiac function) and arrhythmias requiring treatment. Outcomes were assessed prior to discharge from hospital. Secondary outcomes included obstetric, fetal and neonatal events such as (1) pregnancy loss [miscarriage (<20 weeks), stillbirth (>20 weeks’) and neonatal death], (2) fetal growth restriction (estimated fetal weight < 3^rd^ centile for gestational age and sex based on regional population based growth charts or <10^th^ centile with doppler abnormalities, (3) prematurity (birth before 37+0 weeks) and (4) fetal anomalies. In calculating perinatal complications, we used the fetus-at-risk approach [15], i.e. those pregnancies that were terminated prior to the possibility of occurrence of an event were excluded from the denominator while calculating incidences of these events.

### Patient and Public Involvement

Patients or the public were not involved in the design, conduct, reporting or dissemination plans.

### Ethical approval

This study complied with ethical standards set by the Institute Scientific Advisory and Ethical Committee for human studies, in accordance with the 1964 Helsinki declaration and its later amendments. The primary study was approved by the Institute Ethics Committee for human studies (Approval number: JIP/IEC/2016/1079 & JIP/IEC/2019/458). A waiver of consent was obtained for the cases managed between January 2011 and December 2015, and written informed consent was obtained from those who are enrolled thereafter, until August 2020.

### Statistical analysis

Analysis was performed using STATA 15.0 (Stata Corp, Texas, USA). Normality of continuous data was checked with Kolmogorov–Smirnov tests and presented either as mean ± standard deviation, or as median and interquartile range as appropriate. Differences of continuous data between groups were assessed using Student’s t-tests or Mann–Whitney tests. Categorical variables were expressed as frequencies and percentages, and compared using chi-square tests or Fisher’s exact test. Two-sided p-values <0.05 were considered statistically significant.

## Results

We identified 138 pregnancies in 121 women with MHVs, the characteristics of which are shown in Table 1. Ten women had two pregnancies each, two had three pregnancies and one had four pregnancies after valve replacement while on anticoagulation therapy. Valve replacement was undertaken for rheumatic disease in 134/138 (97.1%) pregnancies, and 116 (84.1%) included a solitary MHV in the mitral position. Only 7 (5.5%) valves were of the highly-thrombogenic caged-ball valves. At pregnancy onset, 26 (20%) were in NYHA classes III or IV.

**Table 1:** Characteristics of included pregnancies.

	Pregnancies (n = 138)

Maternal age in years [mean, SD]	25.8 ± 3.8
Nulliparous (n, %)	82 (59.4%)
Acquired (Rheumatic) heart (n, %)	134 (97.1%)
Mechanical (n, %)	
– Ball-and-cage – Tilting disc – Bi-leaflet	7 (5.1%) 124 (89.9%) 7 (5.1%)
Site of mechanical valve (n, %)	
– Mitral – Aortic – Aortic and mitral (n, %)	116 (84.1%) 3 (2.2%) 19 (13.7%)
Age at diagnosis of valvular disease, in years (mean, SD)	14.2 ± 5.6
Age at first valve replacement, in years (mean, SD)	20.6 ± 4.5
Interval between last valve replacement and conception, in years (mean, SD)	5.2 ± 3.8
New York Heart Association (NYHA) class at first visit (n, %)	
– I-II – III – IV	109 (79.0%) 21 (15.2%) 8 (5.8%)
Other comorbidities (n, %)	
– Hypothyroidism – Anaemia – Seizure Disorder	17 (12.3%) 13 (9.4%) 4 (2.9%)

SD = standard deviation, n, % = number and proportion.

Table 2 presents details on anticoagulation. All patients were on VKA prior to pregnancy, and in 14 (10.1%) pregnancies low-dose aspirin was continued during pregnancy. The mean gestational age at the first antenatal visit was 6 weeks, although 11 were seen for the first time >12 weeks and deemed too late to receive counselling regarding sequential treatment; therefore, VKAs were continued. Of the remainder, 106/127 opted for sequential treatment and 21 opted to continue VKAs. Of those on sequential treatment, 4/106 were transferred from other hospitals on weight-adjusted LMWH. The mean daily VKA dose in the first trimester was 3.5 mg warfarin-equivalent and 17 (53.1%) received >5 mg daily. In the sequential treatment group, the mean gestational age for commencing heparins, bridging to VKAs and bridging back to UFH was 6.3 ± 0.5 weeks, 13.0 ± 1.0 weeks and 36.1 ± 1.3 weeks, respectively. Third-trimester bridging to UFH was not possible in 13 pregnancies that presented in preterm labour and underwent unplanned caesarean deliveries. Among the remaining 98 pregnancies, the four that received LMWH in the first trimester, were bridged back to LMWH, while the rest received UFH.

**Table 2:** Details on anticoagulation regimens during pregnancy and the peripartum period (n = 138 pregnancies).

	Sequential treatment (n = 106)	VKA (n = 32)

Anticoagulant used in first trimester	UFH (102, 96.2%) [Mean dose = 21,731 ± 5083 U] LMWH, Enoxaparin (4, 3.8%) [Mean dose = 65 ± 10 mg]	Warfarin (17, 53.1%) [Mean dose = 5.1 ± 1.6 mg] Acitrom (15, 46.9%) [Mean dose = 3.0 ± 0.7 mg]
Gestational age at bridging, in weeks’ gestation (mean, SD)		
• From VKA to Heparin • From heparin to VKA	6.3 ± 0.5 13.0 ± 1.0	NA
Anticoagulant used in second/ early third trimester*	Warfarin (31/94, 33.0%) [Mean dose = 4.9 ± 2.1 mg] Acitrom (63/94, 67.0%) [Mean dose = 3.6 ± 1.1 mg]	Warfarin (12/27, 44.4%) [Mean dose = 5.4 ± 1.8 mg] Acitrom (15/27, 55.6%) [Mean dose = 3.3 ± 0.9 mg]
Gestational age at bridging from VKA to heparin, in weeks/ gestation (mean, SD)	36.1 ± 1.3	36.5 ± 1.0
Anticoagulant in third trimester **	UFH (76/89, 85.4%) [Mean dose = 22,053 ± 4889U] LMWH, Enoxaparin (3/89, 3.5%) [Mean dose = 120 mg] Warfarin (5/89, 5.6%) [Mean dose = 6.7 ± 1.4 mg] Acitrom (5/89, 5.6%) [Mean dose = 2.8 ± 0.5 mg]	UFH (18/22, 81.8%) [Mean dose = 22778 ± 4142U] LMWH, Enoxaparin (1, 4.6%) [Mean dose = 80 mg] Acitrom (3, 13.6%) [Mean dose = 2.7 ± 0.6 mg]
Postpartum transition to VKA, in days (mean, SD)	4.9 ± 1.2	4.5 ± 1.0
Dose of VKA at discharge***	Warfarin (36, 36.0%) [Mean dose = 5.7 ± 2.2 mg] Acitrom (64, 64.0%) [Mean dose = 3.3 ± 1.0 mg]	Warfarin (16, 50.0%) [Mean dose = 6.5 ± 1.8 mg] Acitrom (16, 50.0%) [Mean dose = 3.1 ± 0.8 mg]

LMWH = Low Molecular Weight Heparin; UFH = Unfractionated Heparin; VKA = Vitamin-K Antagonists; NA = not applicable; SD = standard deviation. * After excluding those who had miscarriages and maternal death at <12 weeks of gestation. ** After excluding those who had miscarriage, preterm births and maternal death at less than 28 weeks’ gestation. *** After excluding maternal deaths.

### Primary outcomes (Table 3)

Maternal deaths: There were six maternal deaths (4.3%), all of which occurred in the sequential treatment group. The cause of death in 5/6 cases were thromboembolic complications, while one was secondary to a ventricular arrhythmia. All three antepartum deaths occurred in the first trimester. The first (at 10 weeks’ gestation), was in a patient transferred from another centre on weight-adjusted LMWH, with no prior cardiac events and compliant with treatment. She presented in cardiogenic shock secondary to a valvular thrombus. The second stopped VKA upon confirmation of pregnancy but was seen a week later (at six weeks’ gestation) and had a normally-functioning valve on echocardiography. UFH was commenced at aPTT was therapeutic at 2.5 times the control level, until discharge from hospital at seven weeks’ gestation. She presented at nine weeks’ gestation with sudden-onset breathlessness and was found to be in cardiogenic shock with mitral valve thrombosis, subtherapeutic aPTT and succumbed to a cardiac arrest before repeat valve surgery could be undertaken. The third patient similarly stopped VKA upon confirmation of pregnancy and was referred from a district hospital after commencement of UFH, at 6+3 weeks’ gestation with worsening dyspnoea. Mitral valve thrombosis was confirmed and thrombolysis was started. However, she developed cardiogenic shock and sustained cardiac arrest within two hours of admission. The two other thromboembolic complications occurred on postpartum days three and five. Both patients developed sudden-onset breathlessness and mitral valve thrombi were confirmed. In both these cases, UFH was withheld once in active labour and restarted six hours after delivery, as per protocol. The interval between stopping and restarting the heparin were 12 and 15 hours respectively. In both cases, VKA were resumed on the 2^nd^ postpartum day, and the UFH doses were up-titrated because of subtherapeutic levels of anticoagulation. The patient who succumbed to ventricular arrhythmia was on digoxin and verapamil for atrial fibrillation since conception. She developed new-onset palpitations two days following the preterm birth of a stillborn baby, and could not be resuscitated despite attempting cardioversion.

**Table 3:** Primary (Maternal) Outcomes.

	Sequential treatment (n = 106)	VKA (n = 32)	p value

A. Maternal Death	6 (5.7%)	0	0.34
B. Thromboembolic complications	15 (14.2%)	2 (6.3%)	0.36
C. Hemorrhagic complications – Antepartum haemorrhage – Atonic postpartum haemorrhage* – Surgical site haematoma* – Non-obstetric haematoma	12 (11.3%) 3 5 5 perineal + 1 caesarean wound 1	4 (12.5%) 0 2 2 perineal 0	0.85
D. Cardiac events			
– Heart Failure – Arrhythmia	4 (3.8%) 13 (12.3%)	0 1 (3.1%)	0.26 0.13

VKA- Vitamin K antagonist. * Excluding women who died undelivered(n = 2) and also those who had miscarriage (n = 20).

Thromboembolic complications occurred in 17 pregnancies [15 (14.2%) pregnancies on sequential treatment and 2 (6.3%) on VKAs, p = 0.36]. The five fatal events have been described above, and a detailed account presented in Supplementary Table S1. All events occurred in those with less-thrombogenic (tilting disc and bi-leaflet) valves. Sixteen of these were valvular thrombi, three of which had additional extra-valvular arterial and venous thrombi. One patient presented with left hemiparesis following a thromboembolic stroke involving the middle cerebral artery territory with no evidence of a valvular thrombus. Ten of the 13 antepartum events occurred in the first trimester (6+3 to 10+2 weeks) and in all cases anticoagulation was either subtherapeutic (eight pregnancies on UFH) or, it was (anti-Xa levels) unknown (two pregnancies on LMWH). No thromboembolic events occurred around the time of bridging to VKA or in the second trimester. The three third-trimester events occurred between 34+2 and 36+6 weeks of gestation, following switch over to UFH in view of preterm labour, preeclampsia or as per protocol. In all cases, aPTT values were subtherapeutic. Of the four postpartum cases, three occurred while on UFH (all sub-therapeutic) and one following bridging to VKA, where INR was in the therapeutic range. The non-fatal events occurred on postpartum day three (valvular thrombosis in addition to thrombi in the common iliac and femoral arteries) and day seven (thromboembolic stroke with left hemiparesis). Among 17 cases, 14 including, one who succumbed to mitral valve thrombosis, received thrombolysis and two underwent surgical management – open valve thrombectomy along with caesarean delivery and repeat valve replacement.

Haemorrhagic complications occurred in 16 (11.6%) of all pregnancies [12/106 (11.3%) vs. 4/32 (12.5%), p = 0.85). These included three cases of placental abruption, in patients on sequential treatment. Atonic postpartum haemorrhage occurred in five pregnancies on sequential treatment and two in the VKA group. In addition, eight pregnancies (six in sequential group and two in VKA group) developed surgical site hematomas (seven perineal and one caesarean wound) requiring evacuation and re-suturing. There was one non-obstetrical haemorrhagic event in a patient on sequential treatment, who developed a haematoma in the temple region following a fall, which required surgical evacuation at 37 weeks of gestation.

Cardiac events: There were 18 cardiac events, 17 of which occurred in the sequential treatment group and one (non-fatal arrhythmia) in the VKA group. Of the 17 events in the sequential group, one was a fatal case of arrhythmia and has been described under maternal deaths. The others included four cases of heart failure and 12 cases of non-fatal arrhythmias. There were no instances of infective endocarditis.

### Secondary outcomes

There were 18 spontaneous miscarriages and four pregnancy terminations. All pregnancy terminations were in the VKA group – two following the diagnosis of warfarin embryopathy and two on maternal request for fear of teratogenicity. Both pregnancies diagnosed with warfarin embryopathy were on >5 mg daily doses of warfarin (7.5 mg and 10 mg) in the first trimester. Bilateral renal pelviectasis was noted in four babies (three in the sequential treatment group vs one in the VKA group) and another baby (born to mother on sequential treatment) who succumbed on the third postnatal day had left congenital diaphragmatic hernia. Two neonates in the VKA group succumbed to prematurity-related complications. Apart from early terminations of pregnancy, there were no differences in any of these secondary outcomes between groups, as shown in Table 4. Among pregnancies which progressed beyond 20 weeks, there were 34 cases of fetal growth restriction (29.3%), 26 preterm births (22.4%, in 13 of them bridging was not possible) and five stillbirths (5.4%). Most patients delivered vaginally and caesarean deliveries were reserved for obstetric indications, most commonly fetal distress (n = 14), or when fully anticoagulated on VKAs while in labour (n = 5). However, those babies delivered vaginally since the mothers presented in advanced labour on VKA (n = 8); did not develop any complications. Concurrent caesarean and open valve thrombectomy were successfully performed for a patient who presented with valve thrombosis in cardiogenic shock at 35 +4 weeks gestation.

**Table 4:** Secondary Comparison of obstetrics and neonatal events and complications of pregnant women with mechanical heart valve on anticoagulation.

	Sequential treatment (n = 106)	VKA (n = 32)	p value

Spontaneous miscarriage	13 (12.3%)	5 (15.6%)	0.62
Pregnancy termination	0 (0)	4 (12.5%)	<0.001
Stillbirth^#^	5 (5.4%)	0 (0)	0.58
Neonatal death^##^	1 (1.1%)	2 (8.7%)	0.11
Congenital malformations – all – Warfarin embryopathy	4 (3.8%) 0 (0)	3 (9.4%) 2 (0.06%)	0.35
Birthweight in grams (mean, SD)	2424.7 ± 591.5	2524.8 ± 668.5	0.48
Fetal growth restriction^#^	26 (28.0%)	8 (34.8%)	0.52
Gestational age at delivery in weeks^#^ (mean, SD)	37.0 ± 3.0	36.6 ± 4.6	0.54
Preterm birth^#^	21 (22.6%)	5 (21.7%)	0.93
Preeclampsia	4 (4.5%)	0 (0)	0.58
Gestational Diabetes Mellitus	13 (12.3%)	5 (15.6%)	0.63
Mode of delivery^#^			
• Spontaneous vaginal delivery • Operative vaginal delivery • Caesarean delivery	53 (57.0%) 20 (21.5%) 20 (21.5%)	12 (52.2%) 8 (34.8%) 3 (13.0%)	0.35
Neonatal intensive care unit admission^##^	16 (18.2%)	5 (21.7%)	0.70
Maternal sepsis (puerperal or post-abortal)	5 (4.8%)	2 (6.3%)	0.67
Duration of maternal postpartum hospitalization (median, interquartile range)	10 (8.5)	10 (9)	0.47

VKA = vitamin-K antagonist, SD = standard deviation. ^#^ Includes pregnancies that continued past 20 weeks. ^##^ Excludes spontaneous miscarriages, pregnancy terminations and stillbirth.

Overall, only 84/138 (60.9%) pregnancies were uncomplicated, resulting in the birth of a live, appropriate-for-gestational-age baby at term.

## Discussion

### Main Findings

This study which describes the multidisciplinary management of anticoagulation in 138 pregnancies in women with MHVs in a middle-income country, highlights considerable risk from maternal mortality (4.3%), thromboembolic complications (12.3%), haemorrhagic complications (11.6%), adverse cardiac events (13%), fetal growth restriction (29.3%), preterm births (22.4%), spontaneous miscarriages (13%), congenital malformations (6.5%), stillbirths (3.6%) and neonatal deaths (2.2%). Only 61% of these pregnancies were uncomplicated, resulting in the birth of a live, appropriate-for-gestational-age baby at term. All but one case of thromboembolism was associated with sub-therapeutic anticoagulation and the two cases of warfarin embryopathy occurred in pregnancies where the daily warfarin dose was >5 mg.

### Strength and Limitations

As the randomized controlled trials in the areas are difficult to perform due to the ethical concerns and challenges posed by small numbers of pregnant women with MHVs, observational studies such as this one, form the cornerstone in informing systematic reviews and the determination of optimal anticoagulation strategies for this population. The challenges inherent to LMICs about health care access, compliance, costs and availability of monitoring techniques highlight the importance of reports on the effectiveness and safety of VKA and sequential treatment, which is the focus of this study. Despite limitations that include bias inherent to observational studies, uneven numbers in the two groups; lack of data on compliance, and limited data on aspirin’s role, our study benefits from relatively large sample size and granular details on the pregnancies managed in a middle-income country. Besides, we were able to draw important conclusions to inform practice about anticoagulation strategies in LMICs.

#### Interpretations

Three anticoagulation strategies are considered for pregnant women with MHVs in contemporary practice [5,7,9]. While high-income settings are increasingly using LMWH throughout pregnancy with anti-Xa monitoring [10], its use is challenging in LMICs due to costs, need for anti-Xa monitoring and concerns with compliance.

First, complication rates remain high in this population, despite multidisciplinary surveillance and adherence to protocols [16]. Only 61% of pregnancies were uncomplicated, which is comparable to the global data (58%) reported by the Registry of Pregnancy and Cardiac Disease [17]. However, it was much lower (n =16, 28%) in a cohort 58 women from the United Kingdom Obstetric Surveillance System (UKOSS) data collection system, where 71% of the patients were on LMWH. The maternal death rate was 4.3% in the present series compared to 9% among the cohort report from the UKOSS system [18]. Second, maternal mortality mostly occurs from thromboembolic complications due to subtherapeutic doses of heparin in the first trimester and peripartum period. In addition to UFH in LMICs being administered by patients or personnel with limited training, varying absorption following subcutaneous administration and the destruction by the placental heparinase enzyme, especially in the third trimester, may lead to subtherapeutic levels [19,20]. Compliance with parenteral therapy also remains a challenge due to unfounded fears of fetal harm. Third, therapeutic anticoagulation in pregnancy is associated with haemorrhagic complications [9,21], emphasizing the importance of close titration and cautious re-initiation of anticoagulants in the peripartum period. Fourth, although most cardiac events were non-fatal, they affected many pregnancies, emphasizing the importance of multidisciplinary management in a tertiary referral centre. Fifth, although the two cases of warfarin embryopathy were associated with >5 mg daily doses of warfarin, this dose-dependent association was previously described [22,23]. Fetal complications can occur even with daily doses <5 mg [9,24], not just from warfarin embryopathy, but also from ‘warfarin fetopathy [25]’. Sixth, obstetric complications such as preterm birth and fetal growth restriction are high in LMICs [21], and since the use of low-dose aspirin (75–162 mg/day) can reduce their incidence [26,27], consideration should be given to administering low-dose aspirin to all women with MHVs in LMICs, along with anticoagulation. Finally, challenges inherent to LMICs pertaining to healthcare access, compliance, costs and availability of monitoring techniques emphasizes the need to consider VKA and sequential treatment as valid options for anticoagulation.

## Conclusion

Pregnancy complications associated with MHVs in LMICs may be reduced by multidisciplinary surveillance, avoiding first-trimester warfarin if the daily dose is >5 mg, ensuring therapeutic levels of heparin in the first trimester and peripartum period, and administering low-dose aspirin to prevent placentally-mediated complications of pregnancy.

## Data Accessibility Statement

The data underlying this article will be shared on reasonable request to the corresponding author.

## Additional File

The additional file for this article can be found as follows:

10.5334/gh.1011.s1 Table S1. Details of thromboembolic complications.
