# Supplementary material for: Alternatives to Low Molecular Weight Heparin for Anticoagulation in Pregnant Women with Mechanical Heart Valves in Middle-Income Countries: A Cohort Study
Source: Glob Heart. 2021 Oct 13;16(1):68. doi: 10.5334/gh.1011 (PMC8516007; doi:10.5334/gh.1011)
Supplement: Table S1. — Details of thromboembolic complications. [file gh-16-1-1011-s1.pdf]

Table S1: Details of thromboembolic complications.

Pregnancy number	Valve Type	Assigned anticoagulant regimen	Site of clot	Gestational Age (Weeks)	Anticoagulant at diagnosis	Anticoagulation therapeutic	History of arrhythmias	Final Outcome
**1**	**Tilting Disc**	**Sequential**	**Mitral Valve**	**6+3**	**UFH**	**No**	**No**	**Thrombolysis Death**
2	Tilting Disc	Sequential	Mitral Valve	6+4	UFH	No	Atrial fibrillation	Thrombolysis Discharged
3	Tilting Disc	Sequential	Mitral Valve	6+4	UFH	No	Atrial Fibrillation	Thrombolysis Discharged
4	Tilting Disc	Sequential	Mitral Valve	7+5	LMWH	Unknown	No	Thrombolysis Discharged
5	Tilting Disc	Sequential	Mitral Valve	8	UFH	No	No	Thrombolysis Discharged
6	Bileaflet	Sequential	Mitral Valve, Femoral vein	9	UFH	No	No	Thrombectomy & Valve Replacement Discharged

7	Tilting Disc	Sequential	Mitral Valve	9+1	UFH	No	No	Thrombolysis Discharged
**8**	**Tilting Disc**	**Sequential**	**Mitral Valve**	**9+1**	UFH	**No**	**No**	**Death**
**9**	**Tilting Disc**	**Sequential**	**Mitral Valve**	**10**	**LMWH**	**Unknown**	**No**	**Death**
10	Tilting Disc	Sequential	Mitral Valve	10+2	UFH	No	Atrial Fibrillation	Thrombolysis Discharged
11	Tilting Disc	Warfarin	Mitral Valve,	35+4	UFH	No	No	Open valve thrombectomy with concurrent caesarean Discharged
12	Tilting Disc	Sequential	Mitral Valve External Iliac artery	34+2	UFH	No	No	Thrombolysis Discharged
13	Tilting Disc	Sequential	Mitral Valve	36+6	UFH	No	No	Thrombolysis Discharged

14	Bi-leaflet	Sequential	Mitral Valve, Common Iliac and Femoral artery	Postpartum Day 3	UFH	No	No	Thrombolysis Discharged
**15**	**Tilting Disc**	**Sequential**	**Mitral Valve**	**Postpartum Day 3**	UFH	**No**	**No**	**Death**
**16**	**Tilting Disc**	**Sequential**	**Mitral Valve**	**Postpartum Day 5**	UFH	**No**	**No**	**Thrombolysis Death**
17	Tilting Disc	Warfarin	Thromboembolic stroke involving left middle cerebral artery territory, with left hemiparesis	Postpartum Day 7	Warfarin	Yes	Atrial Fibrillation	Managed conservatively with continuation of VKA Discharged

UFH Unfractionated Heparin; LMWH Low molecular weight Heparin.
